# “Hope is strong”: a qualitative inquiry into serious illness conversations for patients living with structural vulnerabilities and substance use disorders

**DOI:** 10.1186/s12904-025-01893-1

**Published:** 2025-11-17

**Authors:** Emma Schon, Ruth MacRedmond, Rebecca Rechlin, Kelsey Antifaeff, Wallace Robinson, Rose Hatala

**Affiliations:** 1 Division of Critical Care, Department of Medicine, University of British Columbia, Vancouver, BC Canada; 2 School of Nursing, University of British Columbia, Vancouver, BC Canada; 3 Regional Palliative Approach to Care Education (RPACE), Vancouver Coastal Health, Vancouver, BC Canada; 4 Social Worker, Providence Health Care, Vancouver, BC Canada; 5 Division of Palliative Care, Department of Medicine, University of British Columbia, Vancouver, BC Canada

**Keywords:** Acute care, Chronic illness, End-of-life care, Qualitative research, Structural vulnerability, Substance use disorder

## Abstract

**Background:**

A serious illness conversation elicits a patient’s wishes, goals and values in the setting of advancing illness. Patients living with structural vulnerabilities and substance use disorders encounter barriers to these conversations and therefore often experience less than ideal deaths. This study aims to understand the needs and preferences of these patients during serious illness conversations in the acute hospital setting and to inform best practice recommendations for serious illness conversations with this patient population.

**Methods:**

We performed a qualitative research study using interpretive description methodology at a single tertiary care inner-city hospital in Vancouver, British Columbia, Canada. Data was collected from 16 hospitalized participants living with structural vulnerability, substance use disorder and chronic illness. Semi-structured interviews were recorded, transcribed and analyzed iteratively by our research team using thematic analysis.

**Results:**

Participants had unmet basic needs and therefore unique priorities during serious illness conversations. Participants frequently had previous negative healthcare experiences which resulted in them guarding their feelings from healthcare providers. Hope was emphasized as an important component of serious illness conversations. Participants also outlined specific preferences and recommendations for healthcare providers engaging in these conversations.

**Conclusions:**

Our findings offer several important considerations for engaging in serious illness conversations with patients living with structural vulnerabilities and substance use disorders that, if implemented, should improve the quality of conversations and care.

**Supplementary Information:**

The online version contains supplementary material available at 10.1186/s12904-025-01893-1.

## Background

Serious illness conversations are advance care planning communication interventions focused on understanding the goals, values, and wishes of a patient with a serious illness to inform care decisions and ultimately enhance their quality of life [1, 2]. Serious illness conversations improve patient care by reducing unwanted hospitalizations near the end of life, increasing access to palliative care supports and reducing anxiety and depression [1–3]. The Serious Illness Conversation Guide is a tool for healthcare professionals that outlines the flow of conversations and offers specific prompts [4, 5]. Patients living with structural vulnerabilities and substance use disorders experience challenges engaging in these conversations, and there is limited research on how to improve these conversations [6]. Our study sets out to understand the needs and preferences of these patients during serious illness conversations in the acute hospital setting and to inform recommendations for serious illness conversations specific to this patient population.

Structural vulnerabilities include living in poverty and with precarious housing such as single room occupancies, hotels, shelters, or on the street. Patients living with structural vulnerabilities often experience various forms of inequities (trauma and violence), oppression (racism, classism, colonialism), and stigma (mental health, substance use, (dis)ability) that amplify poor health outcomes and restrict access to healthcare, including palliative care [6–8]. In addition, many patients living with structural vulnerabilities also have a substance use disorder [8]. In Canada, thousands of people experience homelessness every year, and up to 60% of these people have used illicit drugs, highlighting the magnitude of this issue [8]. Literature on the lived experience of palliative care patients in the acute setting highlights pain control as a significant concern at the end of life, an issue that is further compounded in patients with opioid tolerance due to substance use [8, 9]. This population falls through the healthcare cracks, receives care either too late, or not at all, and typically experiences less than ideal deaths due to inadequate pain control or limited locations for end of life care [8, 10]. Given the intersecting vulnerabilities that this population experiences, and the subsequent barriers in accessing appropriate and timely healthcare services, understanding their needs and preferences for serious illness conversations is essential in developing best practice guidelines [10].

Significant challenges have been noted during serious illness conversations with patients living with structural vulnerabilities from the perspectives of both the healthcare providers and the patients [8]. Healthcare providers experience this population as resistant to talking about their disease and the dying process, closed in their communication style, and focused on addressing their immediate concerns, such as substance use to avoid withdrawal, their next meal or finding a safe shelter [8, 11, 12]. Structurally vulnerable patients describe not trusting care providers, not believing that their wishes will be respected, and feeling unwelcome, burdensome, and not worthy of care [8, 12–15].

Existing literature on serious illness conversations with structurally vulnerable patients with substance use disorders has used both qualitative and quantitative methodological approaches, however the former was more common. This research describes engaging these patients in serious illness conversations in an outpatient setting such as shelters, hostels, and clinics, highlighting the importance of pre-existing relationships with care providers who have experience caring for patients with substance use and homelessness [10, 16, 17]. Many studies emphasize the importance of treating patients with respect, dignity, and kindness in order to build trust, however, these trusting relationships often take time to build [16, 17]. In the acute hospital setting, serious illness conversations are necessitated by the change in illness trajectory leading to inpatient admission, however, are made increasingly challenging due to time constraints, and the busy hospital environment [18–20]. Furthermore, these patients often seek medical care at later stages of their diseases [16]. There is thus a gap in how to best engage with structurally vulnerable patients in this acute setting, where conversations often occur, and as a result, many die while receiving aggressive interventions and miss the benefit of a palliative approach to their care [20, 21].

The goal of this study is to (1) understand how serious illness conversations unfold between health care providers and patients living with structural vulnerabilities and substance use disorders, and (2) learn the needs and preferences of these patients during these conversations.

## Methods

Our study follows the principles of qualitative interpretive description, which acknowledges the contextual nature of the human experience [22, 23]. Interpretive description aims to capture the subjective experience of participants and therefore requires researchers practice reflexivity and are aware of how their values and experiences may impact outcomes [22, 23]. Our primary goal was to document a rich description of the experiences of patients living with structural vulnerabilities and substance use disorders engaging in serious illness conversations in the acute care setting and identify common needs, preferences, and patient-guided recommendations [24]. Interpretive description allows the discovery of shared experiences amongst participants and is therefore an appropriate methodological approach for achieving our goal [22].

### Setting

Our research took place in the acute setting, on inpatient medical wards at a large urban inner-city hospital in Vancouver, British Columbia, Canada. At our centre, we have the unique privilege and experience of caring for one of the largest concentrations of patients in Canada who experience the intersection of structural vulnerabilities and substance use disorder.

### Reflexivity

Throughout the research process, we recognized that each team member played a role in the construction of knowledge [24]. RR is a research assistant with research experience in goals of care, palliative care and brain health. She brings experience with data analysis with an outsider (non-clinical) perspective. WR and KA are both social workers who have experience engaging with patients who live with substance use disorders, homelessness and other vulnerabilities in Vancouver’s downtown eastside. WR conducted patient interviews and brings the perspective of a social worker with decades of experiences working with this population. KA participated in literature review and contributed expertise in both addictions medicine and social work. RH is a palliative care physician with extensive experience having serious illness conversations in the palliative care setting, as well as a senior researcher and leader in qualitative research projects. She contributes the perspective of a palliative care physician to data analysis and offers expertise in qualitative research. RM is a senior critical care physician with extensive experience having serious illness conversations in the critical care setting, as well as involvement in institution level policy on advance care planning. She contributes the perspective of a critical care physician to data analysis. ES is early in her career in critical care with growing experience in serious illness conversations and end of life policy and education. She conducted patient interviews as well as data analysis from the perspective of a critical care physician.

### Participants

Participant recruitment occurred in person on the inpatient medical units. Enrolment was guided by two main considerations; (1) if clinicians answered ‘no’ to the question, “would you be surprised if this patient died during this hospital admission or in the next 6–12 months?” and (2) if the participant had a code status that was documented as a level of care other than “Full Code” [25]. Both these factors implied that the participant should have had a serious illness conversation and therefore could provide a meaningful interview. Participants met the following general inclusion criteria: receiving care from the addiction medicine consult team, living with a structural vulnerability (related to housing), and experiencing chronic illness. See supplemental appendix 1 for detailed inclusion criteria. A physician member of our research team reviewed the addiction medicine consult team inpatient list and noted patients meeting inclusion criteria by chart review. These potential participants were then approached by a member of our research team conducting interviews, to determine if they were interested in participating in our study. Written informed consent was obtained at the time of interview. Participants were excluded if they did not speak English (as nuances of their experience may be lost through translation) or were unable to participate in the interview process.

### Interview process

Semi-structured interviews were conducted in person in the participant’s hospital room, between April 2022 and December 2023. Interviews were approximately 20–30 min in duration and were recorded and transcribed verbatim. The interviewers asked open-ended questions about the participant’s experience talking to healthcare providers about their health and medical comorbidities, what was helpful and/or unhelpful, their insights about the conversation, and their suggestions about how to improve serious illness conversations. See supplemental appendix 2 for a final version of interview guide. Participants were remunerated with a cash honorarium of $40 after the interview. Participants were offered the opportunity to review their transcripts. Seven participants (of sixteen total participants) asked to review their transcripts, however, due to participant deaths or lack of reliable phone numbers and home addresses, we were only able to contact one of these participants to review their manuscript.

### Thematic analysis

Data collection and analysis occurred iteratively. Our research team met frequently during data collection to undertake preliminary coding and thematic analysis of the transcripts, which subsequently informed ongoing data collection. The interview guide was modified throughout data collection based on the concurrent data analysis to elaborate our understanding of the identified themes, for example asking questions to further explore the theme of hope. Codes were used to identify linkages in the data across all interviews. Continuous evaluation of our interview guide allowed us to explore relationships and patterns. Data were considered sufficient once the interviews were no longer yielding new themes or ideas. Coded interviews were analyzed by members of our research team and connected codes were organized into larger themes that helped to tell our participants’ stories. We used Miro, a collaborative online whiteboard to visually organize our developing themes and NVIVO (version 1.7.2) to aid in the coding and analysis [26, 27].

## Results

We interviewed 16 participants (4 females and 12 males) with ages ranging from 42 to 70 years old. The median interview duration was 26 min 41 s (range 19 min 26 s – 49 min 02 s). We start by sharing a number of upfront observations that frame our results. Due to the acute care environment, our interviews were often interrupted by nursing assessments, meal tray delivery or pick up, overhead announcements, or other patients in a shared room. The interviews proceeded in fits and starts. We highlight this observation as we suspect it is very similar when healthcare providers try to have serious illness conversations with hospitalized patients. Despite these interruptions, at the end of the interviews, our participants frequently expressed gratitude towards the interviewer for taking the time to listen and hope that their feedback would help improve care for others in the future. This observation highlights how much participants value time and conversation with their healthcare providers about their health, and therefore the importance of the results we have outlined below on how to improve serious illness conversations. A final observation was that some participants lacked an understanding of the outcomes of their serious illness conversation and their documented code status. Despite serious illness conversations occurring, participants did not always understand the details, highlighting the importance of improving our serious illness conversations.

Our rich interview data yielded a number of inter-related themes which we outline in more detail below. In essence, as outlined in the theme “Recognizing Patient Context” below, participants often had a number of unmet basic care needs and negative prior healthcare experiences that resulted in participants being guarded with their feelings during serious illness conversations. When healthcare providers established genuine connections with participants and offered hope, richer serious illness conversations were possible.

### Recognizing patient context

Across many interviews, participants’ immediate needs limited their ability to engage in serious illness conversations. Healthcare providers’ agenda during serious illness conversations is often to communicate prognosis and plans for care, understand their patients’ end-of-life goals and wishes and determine an appropriate code status. However, our participants often had different priorities as evidenced by the flow of our interviews and the experiences participants shared with us. The conversation frequently moved towards the participants’ more immediate needs for housing, financial support, or substance use (cravings or withdrawal avoidance). From one participant’s perspective, their most important need was housing. One participant said, “I’m not going to back to where I am living now… I will not go back there. It’s a horrible place to live, let alone die” [P5]. Note: P# in brackets indicates the participant number.

Also limiting many participants’ ability to engage in the serious illness conversation was a history of negative healthcare experiences. One participant described a previous experience in the intensive care unit, “It’s a pretty dismal place, by the way. It’s like a morgue with the people lying down on the bed dying, I guess, I don’t know. It’s horrible” [P11]. Another participant explained interactions with healthcare providers, “there’s others that are – that seem mad at you or something. You’re taking up the medical system’s time, here again, they seem really mad. You know, angry … like, you’re in jail or something talking to a jail guard that’s not the way you should have a feeling in the hospital” [P1].

Whether related to previous negative experiences in healthcare, their current mental state or characteristics of the healthcare provider attempting to engage in a serious illness conversation, participants shared that they often guard their true feelings from their healthcare providers, thus limiting their engagement in serious illness conversations. One participant shared this advice for healthcare providers, “Take the time to really listen and look the patient in the eyes because what comes out of their mouth may not match what you’re seeing in their eyes. Because I think a lot of the time as a patient, I will just nod my head because even if I don’t understand I’ll just go, OK, yeah … a lot of us hide how we feel” [P7].

### The importance of hope

The concept of hope was prevalent throughout our conversations with participants. Participants described deriving hope from conversations with healthcare providers and from their connections with family. When asked to provide advice about these conversations, one participant described, “Well, I think they should say good luck to you or you know we are on your side. We’re not just here, you’re [not] forgotten. We’re on your side. We would like things to go smoothly and healthy … You know give yourself …some hope really” [P1]. Another participant emphasized, “Well, hope is strong. If you have hope, you got everything. Without hope, you don’t have anything” [P8]. Participants highlighted the importance of empowerment and having choice, whether relating to care plans or treatment options. One participant explained, “just let patients know they have choices” [P1].

Participants also described hope related to their connections with family. One participant said, “Well because it’s the most important thing as a human being, is that when we die, that we’re cared for by someone” [P14]. Another participant expressed, “I want to be able to see my daughter graduate. That’s not going to be in the cards most likely because that’s four years, but I want it. Wanting it is not wrong” [P2]. Despite experiencing the significant loss of friends and family associated with substance use and the opioid crisis, some participants reported feeling motivated by these experiences to make positive change in their community and as a result felt hope for their future, “But the choices that I made then I would like to … I would like to do good now, instead of the evil and the chaos that I caused. I would like to do something right. Right for myself and right for others” [P2].

### Participant recommendations for serious illness conversations

Our interviews helped elicit participant preferences related to various aspects of the serious illness conversation. Recommendations from participants fell into two categories; features of the conversation itself and characteristics of the healthcare providers having the serious illness conversation. Participants emphasized the importance of language, “when they come in and I have a question, they answer it and they put it into laymen’s terms, not doctor terms, and I really appreciate that” [P7]. Participants also expressed preferences around having sufficient time for the conversation as well as a lack of pressure to have the conversation, “Yeah, they can like tell you and then, you know, we’ll give you time to think about it and we’ll talk again, and so I think that’s a good way” [P1]. Another participant said, “They were very nice. They always made sure that I was comfortable at talking about the subject. If I wasn’t – and a lot of times I wasn’t, I didn’t want to talk about it – they would leave it alone and leave me alone because I just didn’t want to talk about it” [P5].

Participants described important characteristics or traits of healthcare providers during these serious illness conversations. They highlighted the importance of honesty and directness from their healthcare providers. One participant described attributes of healthcare providers that allow them to open up, “if they’re straightforward and honest. I mean – and they just, you know, gently let you know, this is how it is. You know, these are your options and, and it’s good that someone that will tell you the truth and inform you, keep you … up to date” [P1]. During our interviews with participants, we attempted to understand how they would determine if they could trust their physicians or healthcare providers. Very few participants could pinpoint or identify specific features or characteristics required for trust. However, more commonly, participants talked about ‘just knowing’. In one interview the participant said, “Well, you can just tell by the way they talk to you” [P16]. Another participant describes more specifically, “…if they understand what you’re talking about and they say stuff to you back it lets you know that they’re listening to you and they’ve got a grasp of what – like a good grasp of what you’re getting at. Then that tells you that they’re paying attention and they give a s***” [P8]. One participant described, “Well… they’re not just doing their job, they’re going that extra mile, you know? You can feel it” [P13]. Participants who trusted their care providers also described a reciprocal sense of caring for their physicians, “I mean, people forget that when a doctor comes in, they’re a person, they have feelings, they have families. So that conversation that they have to have with you, it’s really hard” [P6].

## Discussion

Our study describes the patient experience and therefore provides useful considerations when having serious illness conversations with patients living with structural vulnerabilities and substance use disorders such as understanding the patient context, the importance of hope and patient-guided recommendations.

### Contributions to research

Our study offers important new insights regarding the importance of hope in serious illness conversations with patients living with structural vulnerabilities and substance use disorders. The prominence of hope across our data set encouraged us to speculate as to its importance, as there is limited published data on this to date. Perhaps hope in our patients’ context is similar to the importance of hope for adult patients with life-threatening illnesses such as a malignancy. In a qualitative study on patients diagnosed with hematological malignancies, an important theme was an attempt by patients to balance fear and hope when it comes to navigating challenging, life-altering diagnoses [28]. In a review article, authors argue that we can provide hope to patients for many outcomes, not just a cure for a terminal disease, but that patients may hold hope for quality of life, improved relationships with friends and family, control, avoidance of suffering and a peaceful death [29]. In a study of oncology patients hope was a core requirement for patients at the end of life [30]. Hope may be especially salient for our patient population who have lived under unbearably difficult conditions. This finding is timely in light of the new updates to the Serious Illness Conversation Guide in 2023 which added a question related to hopes [5].

Our research was based in an acute care setting which provides new data on serious illness conversations in this population. Most of the current literature is based on studies conducted in outpatient settings such as clinics, hostels, and shelters [10]. The acute inpatient setting often signals a change in illness trajectory that necessitates a serious illness conversation, and therefore is often where these conversations occur. However, the acute setting at the same time, presents unique obstacles to these conversations. Patients are frequently too unwell to engage in these challenging conversations in a meaningful way. If patients are able to participate in serious illness conversations, they are often experiencing heightened emotions and anxieties in the setting of their hospitalization, which can result in guarding or hiding their feelings. Patients may be inaccessible due to timing of procedures, interventions, medication administration etc. Care providers are often busy, new to the patients and lack the longitudinal rapport and trust that exists in the outpatient setting.

### Implications for clinical practice

If we merge our findings with the structure of the Serious Illness Conversation Guide, the following considerations emerge [5]. When setting up the conversation, healthcare providers should first address the patients’ unmet needs, such as housing or finances. Patients do not have the privilege of thinking and planning for the end of life when their basic needs are not being met. Next, when assessing the patient’s willingness to engage in a serious illness conversation, healthcare providers should recognize that a large majority of this patient population may have had previous negative healthcare experiences that make engagement challenging. Healthcare providers should assess if the patient is willing to engage in the current moment. When sharing information with patients living with structural vulnerability and substance use disorder, healthcare providers should do their best to align themselves with the patient. Speaking honestly and genuinely will help build trust with patients while sharing what is often upsetting news. Next, when exploring patients’ goals, fears and wishes particular attention should also be given to the patients’ hopes. This recent prompt was added to the Serious Illness Conversation Guide, but our results further highlight the importance of this question and extends the conversation to various forms of hope; health-related, family-related, care-related. Finally, as healthcare providers close the serious illness conversation, particular attention should be given to ensuring that the patient truly understands the outcomes of the serious illness conversation [5]. We offer these best practice recommendations for engaging in serious illness conversations with this patient population in order to increase the effectiveness of conversations, enhance connections between healthcare providers and patients, and ultimately improve end of life care for our patients living with structural vulnerabilities and substance use disorders.

### Limitations

Our study is not without limitations. Data was collected from a single centre. The participants were largely inhabitants of Vancouver’s Downtown Eastside, a very unique population of patients experiencing intersectional vulnerabilities. We could not sample all of the possible combinations of intersectional vulnerabilities, so we focused specifically on homelessness and substance use disorder. Enrolment was voluntary, however may have been biased towards individuals who are more willing to share their experiences, either positive or negative, with serious illness conversations. Participants were also given a 40$ honorarium, which may have persuaded participants, not otherwise interested in engaging in an interview, to consent to be involved. Finally, interviews were often conducted on busy inpatient units where privacy may be limited and therefore may have impacted our results.

### Future research

Future areas of research include implementation studies using our patient-guided recommendations for serious illness conversations. Consideration could be given to determining feasibility of these recommendations in the acute inpatient setting, patient satisfaction and patient outcomes. Research could also focus on strategies for educating health care providers on study findings in order to improve awareness of the needs of our patients. Finally, further studies could be conducted looking at combined models of care with social workers, to ensure patients’ basic needs are being met, therefore allowing space for serious illness conversations.

## Conclusion

Our research highlights several considerations for serious illness conversations with patients living with structural vulnerabilities and substance use disorders such as understanding the patient context, the importance of hope and patient-guided recommendations. We make several important patient guided best practice recommendations for serious illness conversations with this population that when implemented, alongside current guidelines for serious illness conversations, should enhance conversations and ultimately improve patient care. Future research on the implementation of these recommendations for serious illness conversations would be valuable for healthcare providers and patients.

## Supplementary Information

Supplementary Material 1.

## Data Availability

Questions regarding the raw data analyzed in this study can be directed to the corresponding author. However, the datasets generated and analyzed during the study are not publicly available as we did not obtain consent for data sharing.
