# Supplementary material for: “Hope is strong”: a qualitative inquiry into serious illness conversations for patients living with structural vulnerabilities and substance use disorders
Source: BMC Palliat Care. 2025 Nov 17;24:292. doi: 10.1186/s12904-025-01893-1 (PMC12625005; doi:10.1186/s12904-025-01893-1)
Supplement: Supplementary file 1 — Supplementary Material 1. [file 12904_2025_1893_MOESM1_ESM.docx]

**Supplemental Appendix 1: Inclusion Criteria**

1. Receiving care from the Addictions Medicine Consult Team at St Paul’s Hospital
2. Currently experiencing structural vulnerability through one of the following categories:
   1. Currently experiencing homelessness (no fixed address)
   2. Vulnerably housed (single room occupancy (SRO) or shelter)
   3. Address in the Downtown Eastside neighbourhood
3. Evidence of significant chronic illness and medical frailty demonstrated through one of the following categories:
   1. Advanced comorbidities:
      - Advanced chronic lung disease
      - Severe congestive heart failure (NYHA Class IV) and not a candidate for advanced therapy
      - Cirrhosis (Child Pugh class C or significant GI bleed) and not a transplant candidate
      - Cancer (metastatic cancer or stage IV lymphoma)
      - Stage 4 or 5 chronic kidney disease with deteriorating health
      - Progressive dementia with functional decline
      - Advanced degenerative neurological disease (ie. Parkinson’s, Multiple Sclerosis)
   2. Previous admission to the ICU (not secondary to drug overdose)
   3. Deteriorating functional status in the preceding 6 months

**Supplemental Appendix 2: Interview Guide**

**1. Have you had a conversation with anyone, on this admission or on a previous admission, about what is important to you and what kind of treatment and care you would want if you were to get sicker?**

- **If YES:**
  - What can you tell me about that conversation?
  - Where was the conversation and do you remember who was there?
  - Tell me what you found helpful about that conversation.
  - Tell me what you found unhelpful about that conversation.
  - How did the conversation make you feel?
  - What do you think the purpose of the conversation was?
  - Were you given a chance to say what’s most important to you?
  - Do you think your needs / preferences will be respected?
  - What advice do you have for health care providers for making these kinds of conversations better?
  - How do family or friends or the people closest to you factor into decisions around your health care?
- **If NO:**
  - I noticed that your code status is DNAR “_____”. Did you know this? Can you tell me how this came about and the conversation you had?
  - If still no, see next question about an ideal or imagined conversation

- **If a patient comments on a previous negative health care experience, ask:**
  - Why do you think this experience happened to you?
  - How has this experience changed how you interact with the health care team?

**2. If a health care provider were to have a conversation with you about what is important to you and what kind of treatment and care you would want if you became sicker…**

- What would you want that conversation to be like?
- What would be most helpful?
- What would you want them to NOT do in that conversation?
- How would you be able to tell you could trust them?
- How would you like the conversation to make you feel?
- What advice do you have for health care providers for making these kinds of conversations better?

**3. Some other patients/interviewees have talked about the importance of hope in these conversations. Is hope something important to you in these conversations?**

- What do you think about that?
- What gives you hope?
- When you interact with your health care providers, how do they give you hope? Do they ever take away/reduce your hope?
- What is your hope for your future?

**4. Living amongst the opioid crisis, you may have experienced/witnessed a lot of death and loss. I know I have as a healthcare worker and it’s really hard and sad. Is it OK for us to talk about that? If yes,**

- How has experiencing these losses affected you?
- Has it changed/influenced how you think about your life?
- Do you think it’s influenced how you think about your healthcare or what you would want if your health were to worsen?
